# Mouse Models of CMML

**DOI:** 10.3390/ijms222111510

**Published:** 2021-10-26

**Authors:** Ekaterina Belotserkovskaya, Oleg Demidov

**Affiliations:** 1 Laboratory of Molecular Medicine, Institute of Cytology, Russian Academy of Sciences, 4 Tikhoretskii Prospect, 194064 St. Petersburg, Russia; belotserkovskaya.ev@gmail.com; 2 Laboratory of Excellence LipSTIC and Label Ligue Nationale Contre le Cancer, 7 Boulevard Jeanne d’Arc, 21000 Dijon, France

**Keywords:** chronic myelomonocytic leukemia, CMML, mouse models

## Abstract

Chronic myelomonocytic leukemia (CMML) is a rare and challenging type of myeloproliferative neoplasm. Poor prognosis and high mortality, associated predominantly with progression to secondary acute myeloid leukemia (sAML), is still an unsolved problem. Despite a growing body of knowledge about the molecular repertoire of this disease, at present, the prognostic significance of CMML-associated mutations is controversial. The absence of available CMML cell lines and the small number of patients with CMML make pre-clinical testing and clinical trials complicated. Currently, specific therapy for CMML has not been approved; most of the currently available therapeutic approaches are based on myelodysplastic syndrome (MDS) and other myeloproliferative neoplasm (MNP) studies. In this regard, the development of the robust CMML animal models is currently the focus of interest. This review describes important studies concerning animal models of CMML, examples of methodological approaches, and the obtained hematologic phenotypes.

## 1. Introduction

CMML is a clonal neoplastic hematopoietic stem cell disorder, characterized by dysplasia, monocytosis, and increased risk of transformation to sAML [1]. According to several population-based studies, about 0.3–0.7 new cases per 100,000 people are reported each year in both the United States and European countries [2,3,4,5]. The incidence was shown to be influenced by age, gender, and race, with higher frequency of CMML in old white males [6]. The prognosis for patients with CMML is still dismal, with the median survival being between 12 and 31 months and the incidence risk of transformation into the secondary acute myeloid leukemia being 20% [7,8,9]. Secondary AML is the main cause of lethal outcomes in CMML patients [10].

**Figure 1:**
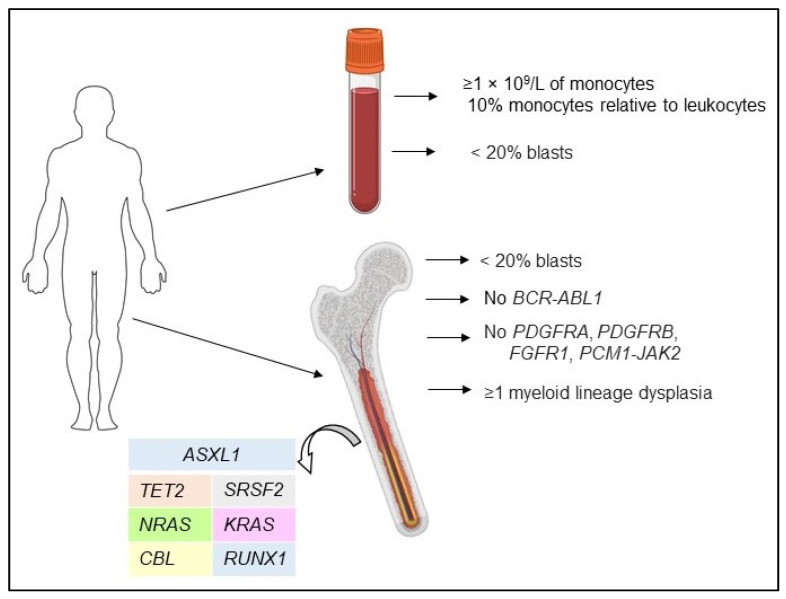
CMML diagnostic criteria.

Originally, CMML was classified by the French–American–British working group as a separate variant of MDS [11]. In 2001, the World Health Organization (WHO) classification defined CMML as a new group, referred to as MDS/MPN (myeloproliferative neoplasm) syndromes, that combines both MDS and MPN features [12]. Diagnosis of CMML is complicated due to overlapping MDS and MPN features, the high heterogeneity of clinical presentation, and the absence of specific indicators for CMML [13]. There are several criteria used in the clinic to diagnose CMML, such as persistent absolute monocytosis (≥1 × 10^9^/L), with monocytes accounting for more than 10% but less than 20% of leucocytes (WBC—white blood cells) in the peripheral blood, dysplasia in one or more bone marrow (BM) cell lineage, and the absence of genetic rearrangements in the *PDGFRA*, *FDGFRB*, and *FGFR1* genes and the *PCM1-JAK2* and *BCR-ABL1* fusions [13,14,15] (Figure 1).

Historically dependent on leukocyte count, CMML is divided into a ‘dysplastic’ variant (MD-CMML) and a ‘proliferative’ variant (MP-CMML) [11]. Individuals with the proliferative type were proven to demonstrate significantly worse outcomes compared those with the dysplastic type [1]. Moreover, these variants of CMML differ in terms of their clinical presentation, gene expression profile and mutational repertoire [16]. The dysplastic variant phenotype develops cytopenias and transfusion dependance, whereas common features of the proliferative variant include leukocytosis, monocytosis, hepatomegaly, splenomegaly, fatigue, night sweats, weight loss, and cachexia [17]. In addition to dysplastic and proliferative types, CMML is subclassified into three variants—CMML-0, CMML-1, and CMML-2—based on the percentage of blasts in the PB and bone marrow [13,18]. These groups are also associated with prognostic significance [1].

Clonal cytogenetic abnormalities are detected in 20–40% of CMML patients. The most common alterations are trisomy 8, loss of the Y chromosome, abnormalities of chromosome 7, complex karyotypes, and the deletion of 20q [7,19,20]. Genomic mutations are detected for the majority of individuals with CMML (>90%) [21]. The most frequent CMML-associated mutations can be categorized as follows: (1) epigenetic modifiers—*ASXL1*, *TET2*, *DNMT3A, IDH1*, *IDH2*, and *UTX* [22,23,24,25]; (2) RNA splicing factors—*SRSF2*, *SF3B1*, *U2AF1*, and *ZRSR2* [26,27]; (3) cell signaling components—*KRAS*, *NRAS*, *JAK2*, *CBL*, and *FLT3* [22,28,29]; (4) transcription factors and nucleosome assembly—*RUNX1* and *SETBP1* [28,30]; (5) tumor suppressor factors—*TP53* and *PHF6* [31] (Table 1).

The highest incidence has been reported for the *TET2* (~60%), *SRSF2* (~50%), *ASXL1* (~40%), and RAS pathway genes (~30%) [13,16]. Most data concerning the prognostic significance of CMML-associated mutations are controversial. Only *ASXL1* mutations have been invariably proven to be independent markers of unfavorable prognosis [22,35]. Interestingly, although the *TET2* effect is ambiguous [42,43], the *ASXL1*^wt^/*TET2*^mut^ combination confers better OS [21]. Although mutations in *NRAS*, *CBL*, *DNMT3A*, and *EZH2* can be considered as determinants of poor prognosis [32,33,50], it is important to emphasize that their lower frequency complicated the correct assessment of their prognostic significance; furthermore, these somatic mutations have been investigated in only a few studies.

In summary, it is worth noting that a growing molecular genetic landscape of CMML made it possible to discover a number of molecular determinants of CMML, but among them there was no specific diagnostic marker of this disease [38].

## 2. Pathogenesis and CMML Treatment

**Figure 2:**
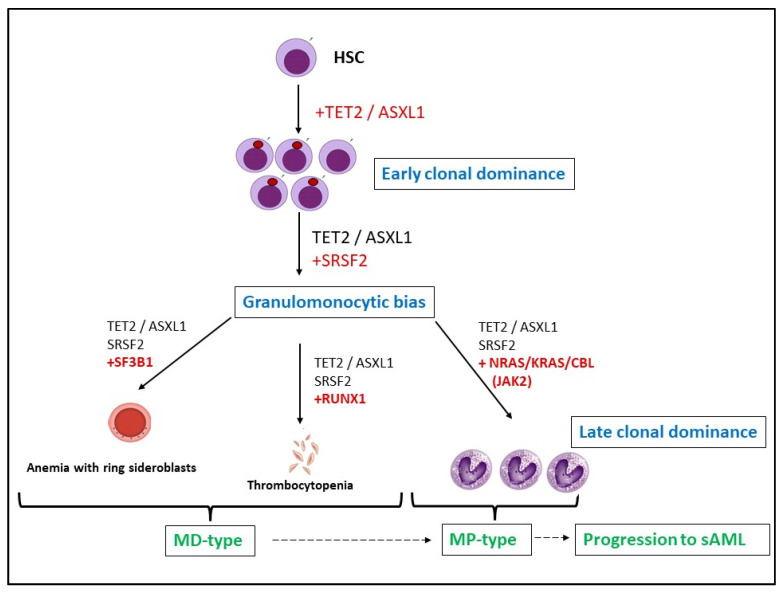
Pathogenesis of CMML. The primary mutations in HSC are *TET2* or *ASXL1*, which promote early clonal dominance. The secondary molecular abnormalities are likely to be associated with spliceosome components, commonly *SRSF2*, resulting in granulomonocytic lineage bias. The third event, which is responsible for late clonal dominance, may involve: (1) *SF3B1* mutations resulting in anemia; (2) *RUNX1*—thrombocytopenia; (3) mutations in *NRAS*, *KRAS*, *CBL*, and *JAK2*—progression of clone. HSC—Hematopoietic Stem Cell; MD-type—myelodysplastic type of CMML; MP-type—myeloproliferative type of CMML.

Although there are increasing amounts of data about the mutational architecture of CMML, the precise scenario of CMML development is still unknown. It is considered that initial events include mutations in *TET2*, *ASXL1,* and *SRSF2* [31,54,55]. Indeed, these mutations are the most frequently cooccurring abnormalities in patients with CMML [56]. Of note, such conditions are similar to clonal hematopoiesis of indeterminate potential (CHIP), a phenomenon characterized by the presence of a clonal blood cell population with neoplasm driver mutations in healthy individuals [57]. Late clonal dominance is suspected to be achieved via the acquisition of mutations in the RAS component pathway, *JAK2*, *SF3B1*, and *RUNX1* [31,54,58], resulting in the dysplastic or proliferative subtypes of CMML (Figure 2). This is consistent with data obtained from patients with CMML, where *SF3B1* and *U2AF1* are associated with the MD-type, while *TET2*, *SRSF2*, *RUNX1*, *NRAS*, *KRAS*, and *EZH2* are the most frequently detected abnormalities for the MP variant [55,59].

It is worth considering the large-scale study conducted by Carr and colleagues [48]. According to whole-exome sequencing data, CMML-associated driver mutations were divided into three groups: (1) primary drivers of chronic CMML and the late transformation stage; (2) mutations of the late transformation phase; and (3) molecular abnormalities that were detected in the chronic CMML phase, but were absent during the late transformation phase. The group of primary drivers included the most abundant mutations in CMML patients, namely *TET2*, *SRSF2*, and *ASXL1*. Predominant members among the late transformation mutations were *NRAS*, *RUNX1*, and *CBL*. Finally, the *NRAS*, *TET2*, and *CBL* mutations were prevalent in the chronic CMML phase, but not in the sAML stage. MD variants of CMML were associated with the splicing mutations and *TET2* (e.g., *SRSF2* and *TET2*). The mutations that activated RAS family members drove the MP variants of CMML, while the *EZH2*, *IDH1/2*, *NPM1*, and *FLT3*-ITD mutations were correlated with acute leukemia transformation. Altogether, this study demonstrates a key role of *RAS* pathway mutations, particularly *NRAS*, in the clonal evolution from CMML (at diagnosis) to sAML [48].

Although modern knowledge about the molecular pathogenesis of CMML has many ‘blind spots’, it was reported that the main features of the CMML clonal landscape include early clonal dominance, the stepwise acquisition of mutations, restricted branching, and the selective advantage of greater numbers of mutated cells during late clonal dominance [31].

Generally, the treatment strategies for CMML are poorly defined and clinical trial data from MDS and other MNPs studies have been adopted for CMML [60,61]. The low incidence of CMML complicates clinical trials involving CMML patients as a distinctive group; in most cases, patients with CMML are explored as a part of an MDS group.

As for MDS, the only curative approach for patients with CMML is considered to be allogeneic hematopoietic stem cell transplantation [62,63]. This therapeutic option is preferable for younger patients with high-risk features [64]. Noncurative therapeutics can be divided into three main treatment directions: hypomethylating agents [32], cytoreductive therapy, and supportive care. The most common drugs for CMML continue to be HMAs, including azacytidine and decitabine [64]. HMAs have been reported to demonstrate low complete response rates and nondurable results, which constitutes controversial data regarding overall survival [32,65]. Many studies have been conducted to reveal the molecular genetic determinants that could predict the response to hypomethylation therapy for CMML, but the obtained data are equivocal (Table 1).

Historically, cytoreductive therapy with hydroxyurea has been considered to be preferable for CMML with proliferative features [60,66]. Supportive therapy focuses on the treatment of anemia, mainly by using erythropoiesis-stimulating agents (ESA) and transfusions [64].

A number of new therapeutic options are being tested for CMML treatment, including: JAK2 inhibitors (ruxolitinib) [67]; inhibition of the RAS family proteins (tipifarnib and NCT02807272) [68]; spliceosome inhibitors (H3B-8800 and NCT02841540); sonic hedgehog pathway inhibitors (glasdegib and NCT02367456); second-generation HMA (NCT02907359)-immunomodulatory agents such as neutralizing antibodies for GM-CSF (Lenzilumab and NCT02546284) and interleukin-3 receptor (CD123) antibodies (Tagraxofusp and SL-401) [69]; lenalidomide [70]; and new medications for supportive care, namely sotatercept [71,72].

In summary, insufficient understanding of the pathogenesis of CMML, including the prognostic significance of CMML-associated genes and the therapeutic response to CMML, which modulates this type of oncohematological disorder in laboratory animals, could help to better comprehend this neoplasm and to develop new, more efficient therapeutic strategies.

## 3. Genetic Models

### 3.1. Oncogenes

Taking into consideration the role of GM-CSF in the pathogenesis of CMML, several groups have created mouse models that recapitulate the activation of the downstream signaling of CM-CSF. GM-CSF is capable of several signaling pathways, including the RAS-MAPK and JAK2-STAT5 pathways [73,74]. Several mouse models of myeloproliferative disorder were created using RAS activation as an initiation event. NF1 is a negative regulator of RAS signaling. The *NF1* knockout mice are embryonically lethal, but the transplantation of fetal mouse *Nf1*^−/−^ cells leads to myeloproliferative disorder in wild-type recipients [75]. Interestingly, the leukemogenic phenotype of transplanted cells in this model could be blocked via the genetic ablation of GM-CSF in both donor cells and recipient mice [76].

Alternatively, CMML can be modeled via the direct genetic modification of RAS proteins. More than 90% of mice transplanted with mutant *Nras*^G12D/+^ bone marrow cells developed MP-CMML-like phenotypes [77,78]. Moreover, the double alleles *Nras*^G12D/G12D^ bearing mice developed MP-CMML phenotypes much more rapidly than *Nras*^G12D/+^ mice, indicating that the incremental activation of Ras signaling is a pathological mechanism that contributes to the development of CMML [79].

The predominant phenotype identified in mice with another constantly activated GTPase, Kras^G12D^, was a myeloproliferative disorder characterized by leukocytosis, splenomegaly, and myeloid hyperplasia in the bone marrow. These mice died during the first two months after birth. Conditional expression of oncogenic *K-ras* from its endogenous promoter in the hematopoietic system induces a lethal myeloproliferative disease in mice, but not AML, indicating that additional mutations are required for the development of AML [80].

It is interesting to note that the mutant Ras models of CMML could be pushed further to enhance the extent of the role of the myeloproliferative phenotype in the development of AML by means of the addition of cooperating mutations to the mouse genome. About one-third of *Dnmt3a*^−/−^; *Kras*^G12D/+^ mice demonstrated an AML-like phenotype that was characterized by the accumulation of immature myeloblast cells in the spleen. Similarly, one-third of *Dnmt3a*^+/−^; *Nras*^G12D/+^ mice developed AML-like phenotypes [81]. Melo-Cardenas J. and co-authors reported that deubiquitylase ubiquitin-specific peptidase 22 (USP22) tissue-specific knockdown in HSCs of Kras^G12D/+^ mice resulted in the rapid occurrence of AML symptoms. USP22 protects an important hematopoietic factor, PU.1, from ubiquitination and subsequent degradation. USP22 deficiency in mice with Ras mutations prevents myeloid differentiation, which may promote the rapid onset of AML [82]. This differs from JMML, in which the deregulation of Ras signaling is a central theme [83].

Another transgenic mouse model, developed to mimic CMML, involved the *Cbl*^Q367P^ knockin of *Cbl*-null mice and the subsequent transplantation of BM cells from these mice to syngeneic recipient mice. CBL is an E3 ubiquitin ligase that negatively regulates β-catenin signaling and several receptor tyrosine kinases. Similarly, to mutations in the RING finger domain in patients with CMML, the *Cbl*^Q367P^ mutation in mice affected the RING domain and abrogated E3 ubiquitin ligase activity, which is essential for the proper functioning of protein. Analyzing *Cbl*^Q367P^ mice, Nakata Y and co-authors found that the PI3K-AKT and JAK-STAT pathways were constitutively activated in long-term hematopoietic stem cells (LT-HSC). In addition to the activation of classical CMML signaling, oncogenic GTPases were deregulated and, together with the overexpression of the *EVI1* transcriptional factor, were found to promote the transformation of CMML to AML [84].

Of note, *RAS* pathway mutations and *CBL* molecular abnormalities are common features in the pathogenesis of JMML [83,85]. In fact, all of the mice mentioned above developed models that were more similar to JMML than to CMML, demonstrating the rapid transformation of chronic disease into acute leukemia.

### 3.2. Epigenetic Regulators

The epigenetic regulators are frequently mutated in CMML. Therefore, several CMML models were created by genetic manipulations with epigenome-controlling genes.

Ten-Eleven-Translocation-2 (TET2), an enzyme involved in DNA demethylation, was found to be altered in nearly half of CMML cases [42]. The mutations in *TET2* are considered by many investigators as the initial event in the development of CMML. TET2 deficiency dramatically reshapes the global pattern of DNA methylation and results in gene silencing. *Tet2* knockout mice are fertile and develop a phenotype that resembles characteristics of CMML at 2–4 months of age. The homozygous *Tet2* deletion distorted the blood formula, which was found to be marked by severe neutrophilia and monocytosis. A necropsy of *Tet2*^−/−^ mice also showed that they had increased BM cellularity, splenomegaly, and a moderately enlarged liver [86]. Eric Solary’s group made an interesting observation, namely that a small single-stranded non-coding RNA, the hsa-miR-150 microRNA, is down-regulated in CMML monocytes in humans. Even though genetic ablation of the hsa-miR-150 analog in mice did not generate a CMML-like phenotype, Mir150^−/−^ mice showed an abnormal monocyte subset repartition. The effect was TET3-dependent, indicating that the TET family of 5-methylcytosine dioxygenases is important in the pathogenesis of CMML [87].

Bera R. and co-authors found that gain-of-function mutants of *ASXL1* (26% of CMML cases) frequently coexisted with a loss-of-function *Runx1* mutation (31% of CMML cases). The transplantation of double mutants, ASXL1-R693X and RUNX1-R135T, with bone marrow cells in recipient mice caused leukocytosis and the detection of dysplastic myeloid cells in peripheral blood, bone marrow, and spleen. At 9 months after transplantation, the mice died with marked splenomegaly and hepatomegaly [88].

Besides the most prevalent molecular epigenetic abnormalities in CMML, such as *TET2* and *ASXL1* mentioned above, CMML can be modeled by manipulations with several other epigenetic genes that are rarely detected in CMML patients but make it possible to reconstitute similar disease phenotypes in mice.

KDM6B (JMJD3) is an epigenetic modulator that positively regulates the transcription of innate immune and developmental genes involved in the pathogenesis of CMML by modulating the methylation status of H3K4 and H3K27. *KDM6B* overexpression alone led to mild hematopoietic phenotypes, but the stimulation with pro-inflammatory agents (LPS or TLR pathways) resulted in significant hematopoietic defects and recapitulated features of CMML [89].

Interestingly, the loss of another member of this family of epigenetic regulators, KDM6A (*Utx*), rather than its overexpression, recapitulates the CMML phenotype in mice. KDM6A also demethylates H3K27 and participates as a subunit in the MLL3/4 H3K4 methyltransferase complex. This CMML model is characterized by long latency in male mice, with the first sign of disease starting from 10 months of age and with less than 70% penetration. In CMML patients, the inactivation of *KDM6A* mutations frequently coincides with mutations of the p53 tumor suppressor gene. The double deletion of *KDM6A* and *TP53* in mice resulted in shorter latency of the disease and was coupled with the presence of anemia, myeloid dysplasia, and blast forms in the peripheral blood [90].

A distortion in another type of histone modification, histone acetylation, is also involved in the pathogenesis of CMML. Histone acetyltransferase (HAT) HBO1(MYST2) was found fused to nucleoporin-98 (NUP98) in leukemic cells from CMML patients. NUP98, as a fusion partner, stabilizes HAT, thereby making HBO1 constitutively active inside the cell nucleus. Transduction of human hematopoietic stem cells (HSCs) with NUP98-HBO1 fusion induces CMML-specific and oncogenic HOXA9 gene signatures through increased H4K8, H4K12, and H3K14 histone acetylation. C57BL/6 mice transplanted with bone marrow cells harboring the fusion gene NUP98-HBO1 recapitulate the CMML-like monocytosis. On the contrary, the inhibition of the HAT activity of NUP98-HBO1 blocks the CMML-like phenotype in mice [91].

An interesting CMML-like phenotype was reported in *Arid4a* deficient mice by Mei-Yi Wu and others in 2008. ARID4A regulates E2F-dependent transcription through its interaction with the E2F repressor retinoblastoma protein RB. The histone methylation pattern was heavily disturbed in *Arid4a* knockout mice. The epigenetic changes resulted in monocytosis in peripheral blood, splenomegaly, hepatomegaly, and reticulin fibrosis in bone marrow that led to mortality of the mice after 6 months of age [92].

### 3.3. Others Regulators of Cell Death (Bid etc.)

The laboratory of Laurent Delva studied a transcription intermediary factor 1γ (TIF1γ), the gene that plays a role in hematopoiesis, and found that TIF1γ is a tumor suppressor in mouse and human CMML [93]. Although mutant *TIF1γ* is almost not detected in CMML patients, the epigenetic-dependent downregulation of this gene was demonstrated in CMML [93]. No serious abnormalities were observed in mice with conditional deletion of *Tif1γ* in HSCs younger than 6 months old. The elder mice demonstrated a rapidly developing myeloproliferative disorder characterized by the progressive hyperleukocytosis in the peripheral blood and increased infiltration of bone marrow, spleen and liver by monocytes. Ubiquitin ligase Tif1γ binds and promotes the ubiquitination of the Smad family regulator of TGF-β signaling. The long latency of disease initiation in this model can be explained by the time required for HSC to acquire additional mutations due to the increased genetic instability in TIF1γ^−/−^ HSC [94].

BID is a pro-apoptotic protein that belongs to the superfamily of BCL2-like proteins. In addition to involvement in the regulation of mitochondria-dependent cell death, BID is able to amplify caspase 8-dependent proapoptotic signaling from cell death receptors. It was found that 30% of heterozygous *Bid*^+/−^ and 50% of homozygous *Bid*^−/−^ mice developed CMML-like conditions at a very old age (2 years old and above). The peripheral blood displayed anemia, thrombocytopenia, and leukocytosis, with a predominance of monocytes and neutrophils. The leukemic mice were distinguished by hepato- and splenomegaly [95].

## 4. Patient Derived Xenograft (PDX) Models

As mentioned above, the specific molecular genetic markers of CMML have not been revealed. This fact complicates attempts to model CMML through CMML-associated mutations and aberrantly expressed genes. This challenge can be overcome with patient-derived xenograft (PDX) models, which save natural features of tumor samples, obtained from a patient, namely the mutation landscape, cell heterogeneity, and therapy response [96]. The most common issue in the creation of PDX models is the low percentage of engraftment. More recently, some successful attempts to PDX modeling for CMML have been published. Yoshimi and colleagues demonstrated almost 100% engraftment of both the CD34^+^ and mononuclear cells of NSG-SGM3 mice from the bone marrow and peripheral blood of patients with CMML [97]. The completed xenotransplant retained specific features of the original tumor. Moreover, this PDX model made it possible to test the JAK2/FLT3 inhibitor, pacritinib.

An interesting example of the PDX modeling of CMML is the study conducted by Taoka and co-authors. They induced pluripotent stem cells (iPSCs) derived from a patient with CMML and created a drug-testing system. Using the developed testing system, some candidate compounds for CMML treatment were identified, specifically a MEK inhibitor, a Ras inhibitor, and a liposomal clodronate [98]. Another CMML xenograft study found the involvement of the BCL2-related protein MCL1 and MEKs in the apoptosis resistance of monocytes in CMML. The combination of MCL1 and MEK inhibitors normalized apoptosis and reduced the expansion of the CMML tumor in mice [99].

Although robust engraftments of patients’ tumors have been achieved, it was shown that the second transplantation is often not successful [97,100]. To overcome this problem, Kloos and colleagues transplanted mice by CMML *NRAS*-mutated cells transduced with the human oncogene Meningioma 1 (MN1) and amplified the CMML PDX model for five generations. Using the established model, it was demonstrated that simultaneous treatment with azacitidine and the MEK-inhibitor trametinib can be considered as an effective therapy in cases of *NRAS*-mutated CMML [101].

According to the research mentioned above, examination of the polo-like kinase 1 (PLK1) inhibitor volasertib in *Nras^G12D^* mutant CMML-patient-derived xenografts has confirmed the role of PLK1 in RAS mutant MP-CMML as well as the potential efficacy of PLK1 inhibition in this type of CMML [48].

Altogether, PDX modeling is a promising approach for the investigation of CMML pathogenesis as well as the testing of potential candidate compounds. Although the maintenance of this model over the course of several generations is still challenging, PDX’s strong point is the consistency of the results obtained by PDX modeling and clinical trials [99,102].

## 5. Conclusions

The development of CMML animal models is a complicated task due to the variety of CMML phenotypes, the mimicking of other hematologic malignancies, and the absence of unique diagnostic markers of this disease. To date, several CMML models were achieved using manipulations with genes involved in cell signaling (*NRAS*, *KRAS*, *CBL*, *FLT3*), epigenetic regulation (*TET2*), and cell death control (*BID*). Although none of the published CMML models reconstitute the complexity of CMML biology, these models have made it possible to identify new genes that participate in the development of CMML, to study the role of CMML-associated genes in more detail, and to test candidate compounds. Moreover, some of the current CMML models can be used to study the transformation this type of blood neoplasm to sAML. In summary, the current progress in CMML modeling suggests that new robust CMML models need to be developed, which will help to explore its pathogenesis and to create pre-clinical platforms for the testing of candidate drugs.

## Figures and Tables

**Table 1:** The most frequently mutated genes in CMML.

Gene Name	Mutation Frequency in CMML, %	Prognostic Significance	Treatment Response to HMA
*ASXL1*	34–46 [22,23,24,25,32,33,34]	Marker of poor prognosis, decreased OS [22,32,33,34,35,36,37] Increased progression to AML [37] Controversial data concerning leukemia-free survival [22,38,39]	Controversial data about response to HMA [22,32,39,40,41]
*TET2*	32–61 [22,23,24,25,27,32,33,34,42]	Controversial data about prognostic impact [22,25,42,43,44,45] Genotype *ASXL1*^wt^/*TET2*^mut^had a favorable impact on OS [4,21]	No impact on response or survival on decitabine [40,41,45] TET2mut/ASXL1wt–higher CR rate and ORR to HMA, prolonged OS after treatment with HMA [32]
*SRSF2*	29–52 [22,24,27,32,33,34,46]	Controversial data about prognostic impact [22,27,46]	No impact on response to HMAs [22,32,39,41]
*RUNX1*	6–22 [22,24,25,27,32]	Controversial data about OS [32,47] Trend towards increased progression to AML [47]	No impact on response to HMAs [22,32,41]
*NRAS*	2–22 [22,23,24,25,27,32]	Decreased OS [33,48]	No impact on response to HMAs [32,40,41]
*KRAS*	3–12 [23,24,25,27]	Unclear impact on prognosis [38]	No impact on response or survival on decitabine [40,41]
*CBL*	10–22 [22,23,24,25,27,32]	Decreased OS [22,32]	No impact on response [32,40,41] Controversial data about OS after therapy with HMAs [22,40]
*U2AF1*	5–10 [22,24,32]	No impact on prognosis [49]	No impact on response to HMAs [32,41]
*DNMT3A*	2–9 [22,24,32]	Decreased overall survival [50] Decreased leukemia-free survival [50]	No impact on response to decitabine [41]
*SETBP1*	4–18 [13,24,30,34]	Controversial data about OS and its impact on progression to AML [13,30,35,39,51,52,53]	Unclear impact
*IDH2*	4–6 [22,24,25]	Controversial data about prognosis [22,25,38]	Controversial data [22,41]
*EZH2*	5–11 [22,25,27]	Decreased OS, increased progression [25,33,38]	Unclear impact
*FLT3*	<5 [29,38,49]	No impact on prognosis [29,40]	No impact on response to decitabine [40]

OS—Overall Survival; CR—Complete Remission; HMA—Hypomethylating Agent.
